# Molecular Characterization and RNA Interference Analysis of SLC26A10 From *Nilaparvata lugens* (Stål)

**DOI:** 10.3389/fphys.2022.853956

**Published:** 2022-03-17

**Authors:** Ruijuan Zhang, Jinliang Ji, Yabin Li, Jianbin Yu, Xiaoping Yu, Yipeng Xu

**Affiliations:** Zhejiang Provincial Key Laboratory of Biometrology and Inspection and Quarantine, College of Life Sciences, China Jiliang University, Hangzhou, China

**Keywords:** *Nilaparvata lugens*, SLC26A10, RNAi, ovarian development, reproduction

## Abstract

*SLC26A10* is a member of the SLC26 gene family, but its role in insects is still unclear. We cloned the *SLC26A10* gene of *Nilaparvata lugens* (*NlSLC26A10*) and found *NlSLC26A10* contained 11 transmembrane regions and a STAS domain. Expression pattern analysis showed *NlSLC26A10* expression was more upregulated in adults than in nymphs, highest in the ovary. After injection of double-stranded RNA (dsRNA) of *NlSLC26A10*, the mRNA level of *NlSLC26A10* significantly decreased and, consequently, the ovarian development of adult females was hindered; the amount and the hatchability of eggs and yeast-like symbionts in mature oocytes decreased. Further study showed that *NlSLC26A10* might result in decreased juvenile hormone level and vitellogenin expression. These results indicate that *NlSLC26A10* plays an essential role in the reproduction of *N. lugens*.

## Introduction

The brown planthopper, *Nilaparvata lugens* Stål (Hemiptera: Delphacidae), is a devastating insect pest of rice in Asia (Heong and Hardy, 2009). *N. lugens* sucks phloem sap and spreads viruses, seriously harming the plant (Sōgawa, 1982; Zheng et al., 2014). In recent years, outbreaks of *N. lugens* have been continuously recorded, causing 1–1.5 billion kilograms of rice production losses per year in China. As an *r*-reproductive strategy insect, *N. lugens* has a strong adaptability to develop resistance to chemical insecticides or resistant rice varieties, making it hard to be controlled. Therefore, it is urgently needed to study new control strategies of *N. lugens* (Bottrell and Schoenly, 2012). A control strategy is to identify genes that play vital roles in the growth and development of *N. lugens* and use these genes as targets. For example, RNA interference (RNAi) is an effective alternative technique for controlling *N. lugens* based on target genes (Whitfield and Rotenberg, 2015; Bao and Zhang, 2019).

Solute carrier family 26 (SLC26) is a conserved multifunctional anion exchanger family with 11 members (SLC26A1-SLC26A11), involved in anions secretion and absorption. The SLC26 family members contain transmembrane regions and a C-terminal STAS domain (sulfate transporter and anti-sigma factor antagonist; Wang et al., 2019). Most members of the SLC26 family act as anion exchangers, only SLC26A7 and SLC26A9 have been shown to act as anion channels alone. Because the SLC26 family was newly found in recent years, the functions of its members have not been well explored, and only some general features and potential cellular function have been introduced. Members of the SLC26 family have been reported to be associated with several diseases and symptoms, including multiple epiphyseal dysplasia (SLC26A2), congenital chloride diarrhea (Slc26A3), Pendred syndrome (SLC26A4), nonsyndromic (isolated) hearing impairment (SLC26A5), calcium oxalate kidney stones (SLC26A6), congenital hypothyroidism (SLC26A7), male infertility (SLC26A8), and gastric hypochlorhydria (SlC26A9; Sindić et al., 2007; Ohana et al., 2009; Alper and Sharma, 2013). Until to now, the physiological roles of SLC26A1, SLC26A10, and SLC26A11 have not been reported. As a member of the SLC26 family, SLC26A10 (SLC26 member 10) has been reported in mammals, including humans (Mount and Romero, 2004), mice (Alvarez et al., 2004), guinea pigs (Andharia et al., 2018), and Australian sheep (Knight et al., 2012), but its function is still unclear.

In our previous experiment, we found *SLC26A10* was highly expressed in ovaries during a transcriptome analysis of *N. lugens*. This implied that *SLC26A10* might play an important role in the ovarian development of *N. lugens*. In the present study, we cloned *SLC26A10* of *N. lugens*, performed bioinformatics analysis, and examined the expression patterns of *NlSLC26A10* by qPCR. Furthermore, we studied the function of *SLC26A10* in *N. lugens* by RNAi.

## Materials and Methods

### Tested Insects

*Nilaparvata lugens* used for this study were maintained in a climatron at China Jiliang University, Hangzhou, China. The population of *N. lugens* was reared on rice TN1, at 27 ± 1°C, with a 16:8 h (light:dark) photoperiod.

### Extraction of Total RNA and Cloning of *NlSLC26A10*

Total RNA was extracted from *N. lugens* adult females with TRIzol Reagent (Takara, Dalian, China), following the manufacturer’s instructions. After verifying the integrity and measuring the concentration of RNA, 1 μg total RNA was used for cDNA synthesis with a PrimeScript™ II first Strand cDNA Synthesis Kit (Takara, Dalian, China). A pair of primers (*NlSLC26A10*-F and *NlSLC26A10*-R; Supplementary Table S1) was designed with Primer Premier 5.0 software and we amplified the *NlSLC26A10* sequence using Premix Taq™ (LA Taq™ Version 2.0, Takara, Dalian, China). Then, the PCR product was cloned into the pMD™19-T vector (Takara, Dalian, China) and sequenced.

### Sequence Analysis

The open reading frame (ORF) of *NlSLC26A10* was predicted using the ORF Finder. Its isoelectric point (*pI*) and molecular weight (Mw) were predicted with ExPASy.^1^ The signal peptide and transmembrane helices were predicted with SignalP^2^ and TMHMM Server v. 2.0,^3^ respectively. The protein domain was predicted with HMMER.^4^ The *Nl*SLC26A10 protein sequence was compared with other SLC26A10 sequences in the NCBI database.^5^ Multiple sequences were aligned by DNAMAN software. The evolutionary tree was constructed with the neighbor-joining method using MEGA 10.2.

### Real-Time Quantitative PCR Analysis

The mRNA level of *NlSLC26A10* was analyzed by real-time quantitative PCR (qPCR). Total RNA of samples was extracted, and 1 μg total RNA was used for cDNA synthesis with a Perfect Real-Time PrimeScript™ RT reagent Kit with gDNA Eraser (Takara, Dalian, China). Pairs of primers for qPCR analysis of target genes were designed with Primer Premier 5.0 software (Supplementary Table S1), and their specificity was confirmed by the agarose gel electrophoresis and melt curve analysis of the qPCR product. The qPCR was performed in 20 μl reactions, including 10 μl TB Green® Premix Ex Taq™ II (Tli RNaseH Plus; Takara, Dalian, China),1 μl each primer (10 μM), 2 μl cDNA template, and 5.6 μl ultrapure water. The program of qPCR was 94°C for 30 s, and 40 cycles of 94°C for 5 s and 60°C for 30 s. The expression level of *Nl*SLC26A10 was evaluated by the 2^−∆∆Ct^ method, taking the *N. lugens* 18S rRNA (*Nl*18S) as the internal control gene (Wu et al., 2019).

### RNA Interference

A pair of primers (T7-*NlSLC26A10*-dsF and T7-*NlSLC26A10*-dsR) that contained a T7 polymerase promoter was designed for amplifying the DNA template of *SLC26A10* dsRNA (ds*SLC26A10*; Supplementary Table S1; Milligan et al., 1987; Wu et al., 2019). The DNA template was 591 bp; dsRNA of the GFP gene (ds*GFP*) was used as the negative control. The GFP gene sequence was synthesized *in vitro* and referred to as the binary vector pCAMBIA-1,302 (GenBank: AF234298.1). The primers for synthesizing ds*GFP* were T7-*GFP*-dsF and T7-*GFP*-dsR, and the DNA template for ds*GFP* synthesis was 350 bp. The dsRNA was synthesized by an Invitrogen™ MEGAscript™ T7 Transcription Kit (Ambion, Austin, TX, United States), following the manufacturer’s instructions. The integrity of synthesized dsRNA was verified by 1% agarose gel electrophoresis and its concentration of RNA was measured by a NanoDrop 2000 system (Thermo Fisher Scientific, Waltham, MA, United States).

RNAi was performed by dsRNA injection. Newly emerged virgin female adults were anesthetized with ice for 40–50 s and about 50 nl of dsRNA (5,000 ng/μl) was injected into the abdomen using a manual microinjector.

### Dissection Observation and Fertility Analysis

The healthy individual females injected with dsRNA were transferred to fresh rice seedlings in a jar with two untreated male adults. To count the number of eggs, fresh rice seedlings were changed every day until the females died. The survival rate of female adults and the number of eggs (hatched and unhatched) were counted. For dissection, insects treated with dsRNA were anesthetized on ice and then, the cuticle was carefully removed. The tissues in PBS were imaged under a stereo zoom microscope (Nikon SMZ1500, Tokyo, Japan), photographed with NIS Elements software.

### Quantity Statistics of Yeast-Like Symbionts in Mature Oocytes

Yeast-like symbionts (YLSs) are primary symbionts of *N. lugens* (Noda et al., 1995) and are transovarially transmitted in the *N. lugens* population (Houk and Griffiths, 1980). The YLSs enter the oocyte through ovarian epithelial plug and develop into bacteriocytes in the posterior end of the oocyte after oocyte maturation (Cheng and Hou, 2001). The number of YLSs in the mature oocyte of *N. lugens* was counted after dsRNA injection. After dissection from the female adults, the mature oocytes were treated with 35% sodium hypochlorite solution. Then, the bacteriocytes were released from the posterior end of the oocytes, and the YLSs were released from the bacteriocytes. After YLSs were well spread in the solution, they were imaged under a Nikon inverted microscope (ECLIPSE Ti-S, Tokyo, Japan), photographed with NIS Elements software.

### Immunofluorescence Analysis

Immunofluorescence was used to analyze the localization and distribution of target proteins. The immunofluorescence staining was carried out as described previously (Nan et al., 2016). The primary antibody (Anti-SLC26A10) was a rabbit polyclonal antibody against a polypeptide (ENDPLLGNEDSGGKC) of *NlSLC26A10* deduced protein. The specificity of the primary antibody has been verified by Western blot. The secondary antibody was goat anti-rabbit IgG antibody conjugated with Dylight 488 fluorescent dye (Abbkine, California, USA). Samples were imaged and photographed with Laser Scanning Confocal Microscope (Leica SP8, Mannheim, Germany).

## Results

### Identification and Phylogenetic Analysis of *NlSLC26A10*

**Figure 1:**
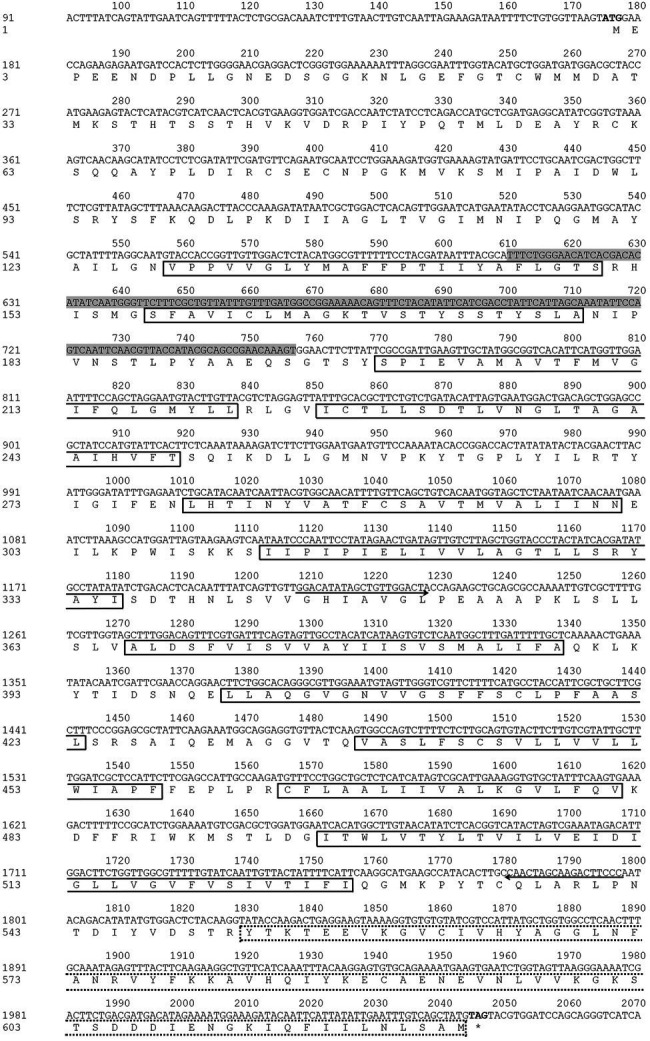
Nucleotide and deduced amino acid sequence of *NlSLC26A10*. The start codon (ATG) and stop codon (TAG) are in bold. The primers used for amplifying the DNA template of dsRNA are shown with direction arrows. The template sequence for qPCR is shown in the shadowed area. The 11 transmembrane regions are shown in full-lined boxes, and STAS (552–623 aa) is shown in a dot-lined box.

**Figure 2:**
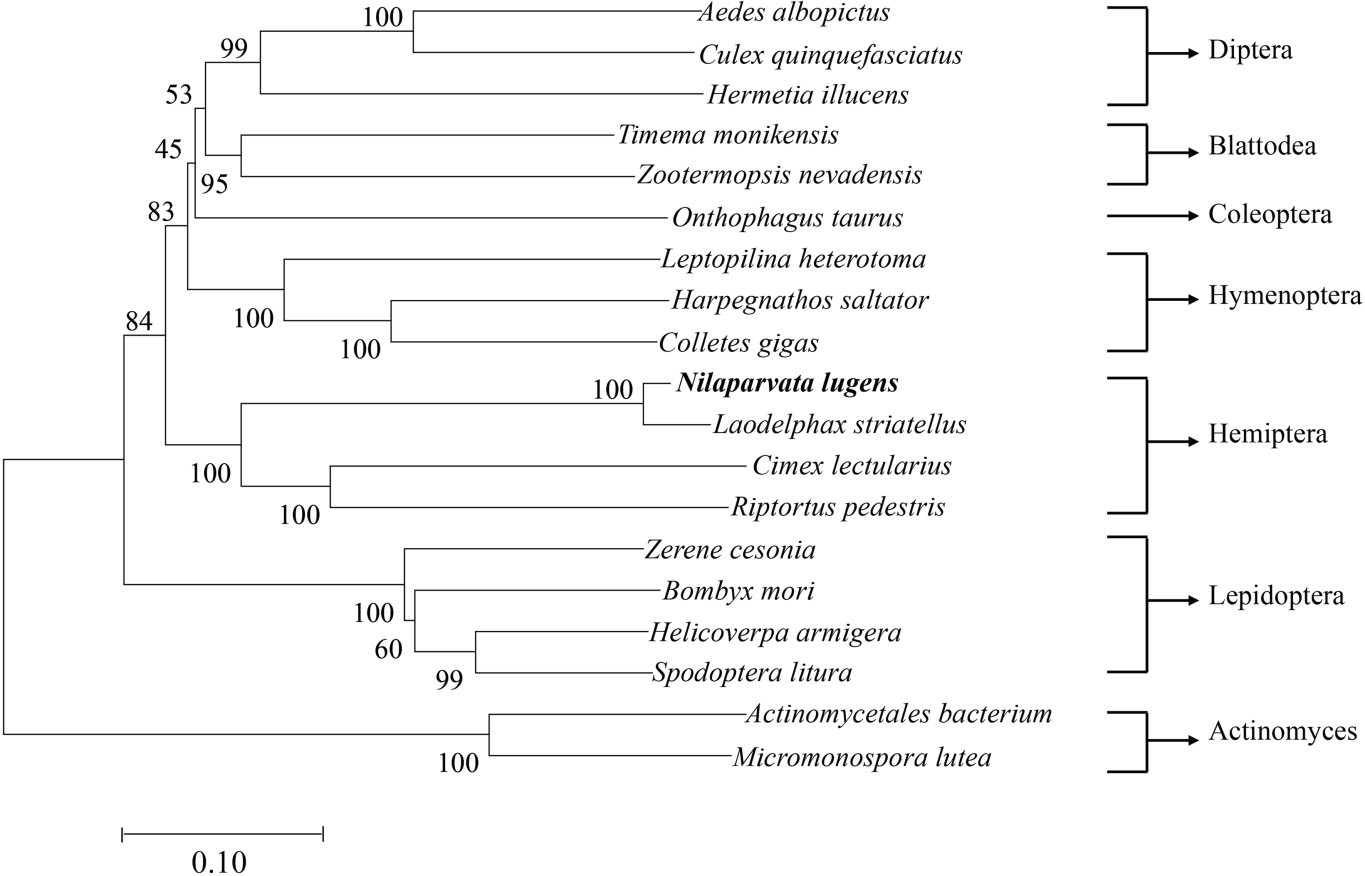
The diagram of phylogenetic relationships of NlSLC26A10. The analysis involved 19 nucleotide sequences, *Actinomycetales bacterium* and *Micromonospora lutea* were used as outgroups. The bootstrap of trees was 1,000 replicates. *Nilaparvata lugens* and *Laodelphax striatellus* are closely related. *Aedes albopictus*, XP 029731531.1; *Culex quinquefasciatus*, XP 038112203.1; *Hermetia illucens*, XP 037917492.1; *Timema monikensis*, CAD7432363.1; *Zootermopsis nevadensis*, XP 021939110.1; *Onthophagus taurus*, XP 022906818.1; *Leptopilina heterotoma*, XP 043475130.1; *Harpegnathos saltator*, XP 011151869.1; *Colletes gigas*, XP 043248168.1; *Laodelphax striatellus*, RZF38072.1; *Cimex lectularius*, XP 014247282.1; *Riptortus pedestris*, BAN20811.1; *Zerene cesonia*, XP 038213017.1; *Bombyx mori*, XP 021205545.1; *Helicoverpa armigera*, XP 021191371.1; *Spodoptera litura*, XP 022831360.1; *Actinomycetales bacterium*, NLT53914.1; and *Micromonospora lutea*, WP 204001039.1.

*NlSLC26A10* has an 1872 bp ORF that encodes a protein of 623 amino acid residues (GenBank: OM282973) with a calculated molecular mass of 68.2 kDa and an isoelectric point (*pI*) of 6.51, without signal peptides. As expected, *NlSLC26A10* contains 11 transmembrane regions and a STAS domain (Figure 1). Sequence comparison and phylogenetic analysis show that SLC26A10s are conserved in animals (Supplementary Figure S1; Figure 2), indicating SLC26A10 has some basic functions in insects.

### Temporospatial Expression Pattern of *NlSLC26A10*

**Figure 3:**
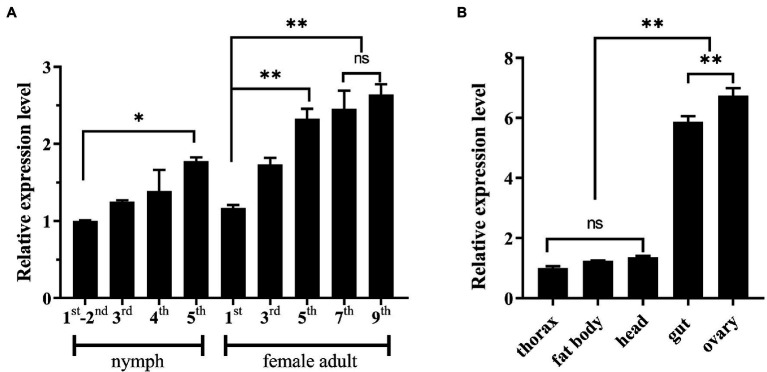
Expression patterns of *NlSLC26A10*. **(A)** Temporal expression pattern of *NlSLC26A10*. Developmental stages include 1–5 instars of nymphs and 1–9 day old female adults. **(B)** Tissue expression patterns of *NlSLC26A10* in five tissues of female adults. All data are presented as the mean ± standard error (Data were analyzed by GraphPad Prism 2.0 and one-way ANOVA). Significant difference: * means *p* < 0.05, ** means *p* < 0. 01, and ns means no significance.

The expression pattern of *NlSLC26A10* was analyzed by qPCR (Figure 3). *NlSLC26A10* was expressed in all developmental stages, and its expression level in nymphs and female adults was higher (Figure 3A). *NlSLC26A10* was expressed in all tested tissues, including the head, thorax, fat body, gut, and ovary. Among these tissues, *NlSLC26A10* expression was highest in the ovary, followed by the gut (Figure 3B).

### Effects of RNA Interference

**Figure 4:**
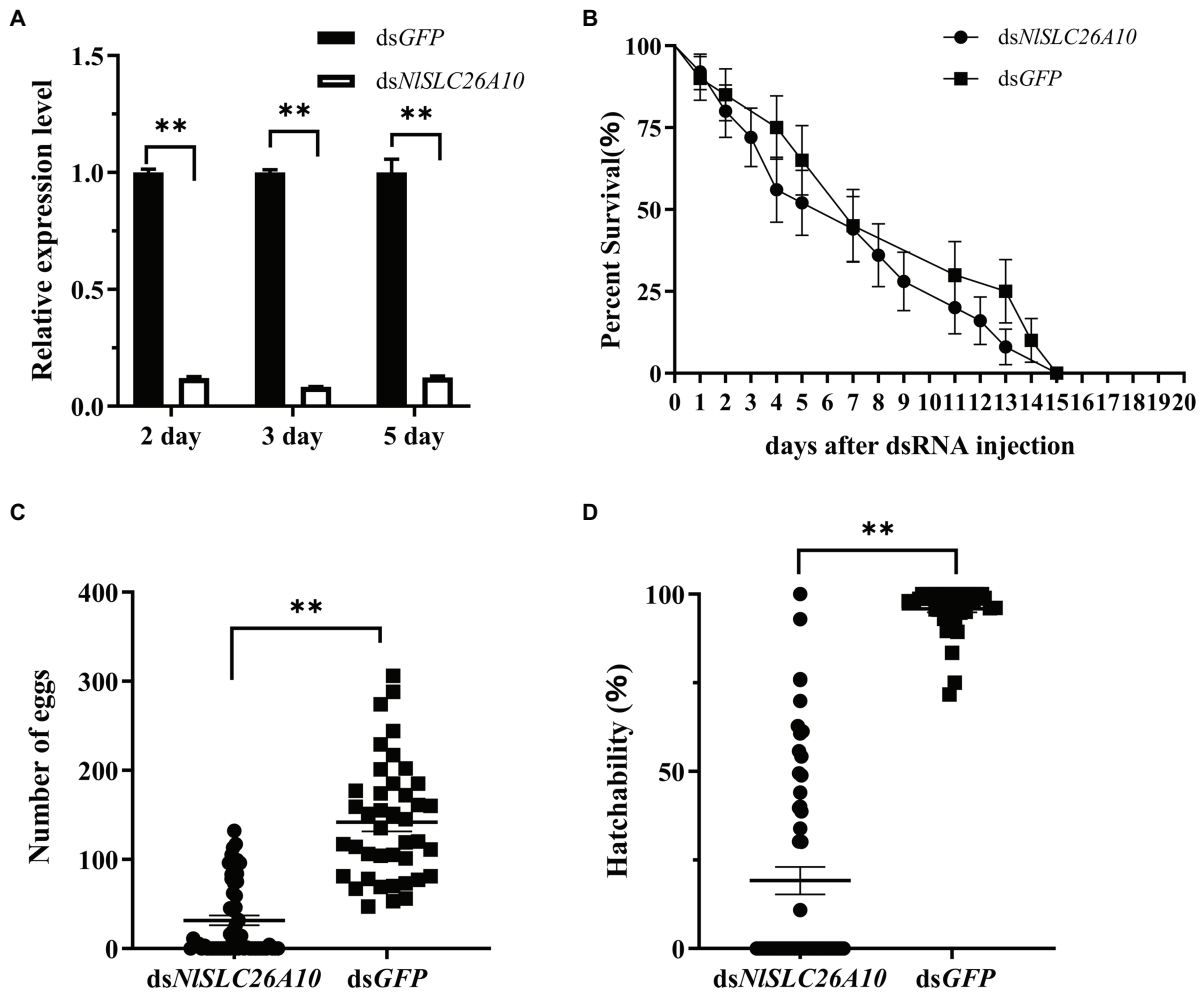
Effects of ds*NlSLC26A10* injection on *N. lugens*. **(A)** The efficiency of RNAi-mediated knockdown of *NlSLC26A10* at 2, 3, and 5 days post-injection of dsRNA. The relative expression level of *NlSLC26A10* in ds*GFP* and ds*NlSLC26A10* group was separately calculated at each day during calculation. **(B)** The survival of *N. lugens* after ds*NlSLC26A10* and ds*GFP* injection (three groups and 15 individuals in each group). **(C)** The total number of eggs laid by ds*NlSLC26A10*-treated (*n* = 56) and ds*GFP*-treated (*n* = 41) females. **(D)** Hatchability of *N. lugens* for ds*NlSLC26A10-*treated (*n* = 56) and ds*GFP*-treated (*n* = 41) females. Data were analyzed by GraphPad Prism 2.0, one-way ANOVA, and unpaired two-tailed *t*-tests. All data are presented as the mean ± standard error. ** in **(A,C,D)** represent significant differences of *p* < 0.01. No significant difference was found in **(B)**.

To detect the interference efficiency of ds*NlSLC26A10*, female adults at 2, 3, and 5 days post-injection of dsRNA (d.p.i.) were collected. qPCR results showed that *NlSLC26A10* expression was significantly downregulated by 87.9, 91.7, and 87.8% at 2, 3, and 5 d.p.i., respectively, showing that RNAi-mediated knockdown of *NlSLC26A10* was effective (Figure 4A).

#### Effects of RNAi on the Survival of *N. lugens*

The number of the living individuals until 20 d.p.i was recorded. The results showed that there was no obvious difference in survival rate between ds*NlSLC26A10* and ds*GFP* treatments (Figure 4B), confirming ds*NlSLC26A10* had no lethal effect on *N. lugens*.

#### Effects of RNAi on the Laying of Eggs

The total number of eggs (hatched and unhatched) laid by ds*NlSLC26A10*-treated and ds*GFP*-treated female adults within 20 days were counted. After ds*GFP* injection, three-quarters of female adults laid more than 100 eggs and the average number of eggs was 142. Injected with ds*NlSLC26A10*, two-fifths of female adults laid no egg and the average number of eggs was 32 (Figure 4C). The hatchability of eggs laid by ds*NlSLC26A10*-treated females was much less than that of ds*GFP*-treated (Figure 4D). These results indicate ds*NlSLC26A10* injection affects the spawning and egg hatching of *N. lugens*.

#### Effects of RNAi on the Number of YLSs

**Figure 5:**
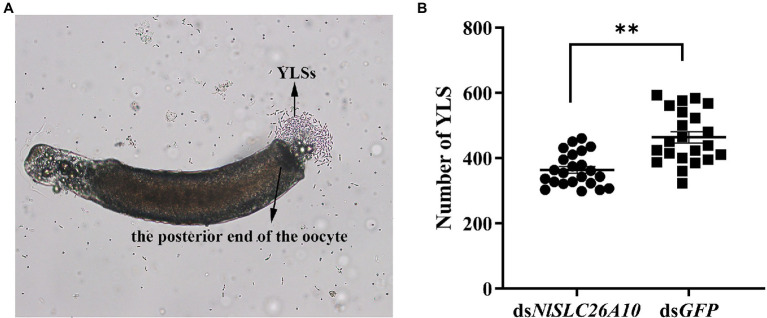
YLSs were released from mature oocytes under the microscope and the number of YLSs in the mature oocytes was counted after dsRNA injection. **(A)** YLSs were released from the posterior end of the oocyte. **(B)** The number of YLSs in the mature oocytes of ds*NlSLC26A10*-treated (*n* = 23) and ds*GFP*-treated (*n* = 22) *N. lugens* females. The YLSs in the budding state were counted as two. Data were analyzed by GraphPad Prism 2.0 and unpaired two-tailed *t*-tests. ** in **(B)** represents significant differences of *p* < 0.01.

In addition, the number of YLSs in the mature oocytes of *N. lugens* at 5 d.p.i. was recorded. The results showed the number of YLSs from the ds*NlSLC26A10* treatment was lower than ds*GFP* (Figure 5), demonstrating that the injection of ds*NlSLC26A10* affects YLSs entering *N. lugens* oocytes.

#### Effects of RNAi on Ovary Development

**Figure 6:**
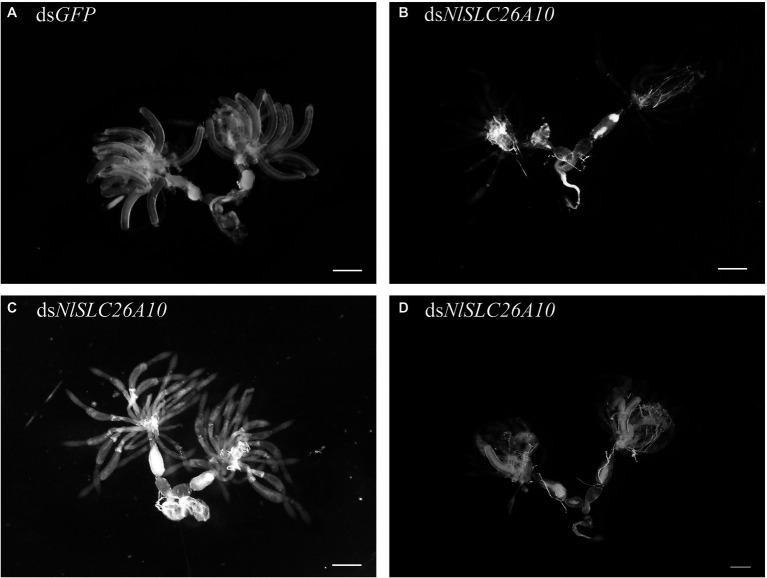
Effects of ds*NlSLC26A10* on the ovarian development of *N. lugens*. **(A)** Ovaries from ds*GFP*-treatment at 5 day post-injection of dsRNA. In **(B–D)**, ovaries were affected to varying extents from ds*NlSLC26A10-*treatment at 5 day post-injection of dsRNA. Scale bar: 100 μm.

The female adults at 1–5 d.p.i. were dissected. Within 1–3 d.p.i., there was no obvious difference in most ovaries between ds*NlSLC26A10*-treated and ds*GFP*-treated. However, the difference was obvious at 5 d.p.i. At this time, ovaries of ds*GFP*-treated were matured, fully developed, and banana-shaped (Figure 6A), but in ds*NlSLC26A10*-treated females, most ovaries were small, poorly developed, had fewer mature oocytes, and remained immature up to 5 d.p.i. (Figures 6B–D). The results demonstrate that *NlSLC26A10* is related to the ovarian development of *N. lugens*.

### Immunofluorescence Analysis of *Nl*SLC26A10 Expression

**Figure 7:**
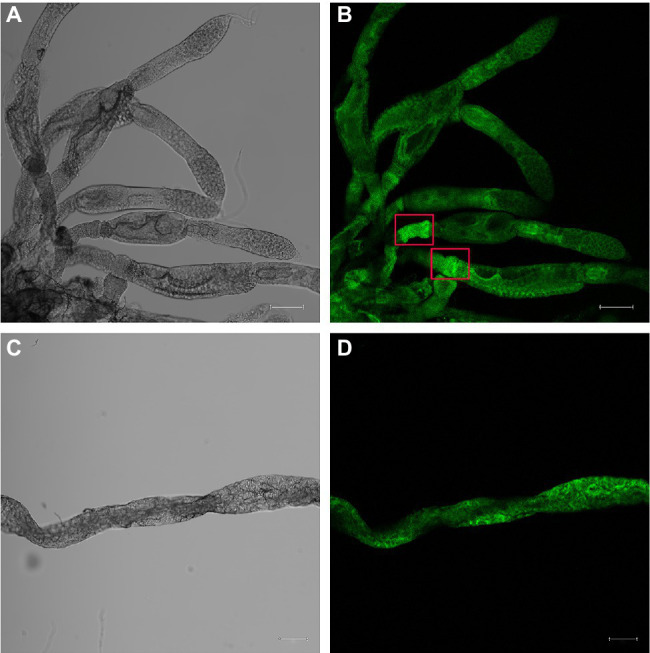
Immunofluorescence analysis of *Nl*SLC26A10 expression in the ovary **(A,B)** and gut **(C,D)** of untreated *N. lugens*. Fluorescence signals are stronger in the ovariole pedicels (red boxes in **B**). The green signal represents the location of *Nl*SLC26A10. The Scale bar: 100 μm.

**Figure 8:**
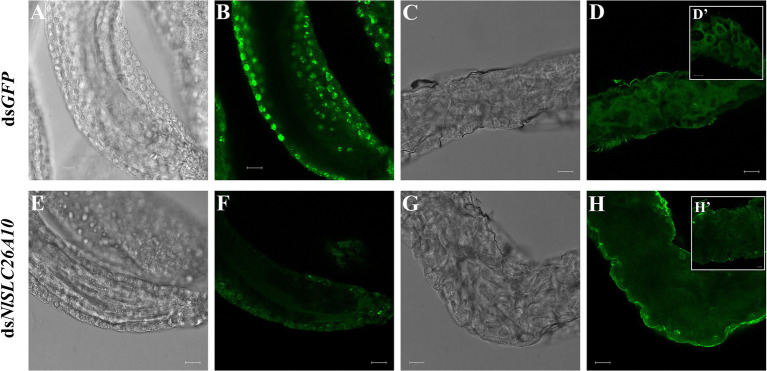
The expression of *Nl*SLC26A10 in the ovarian follicle and gut after RNAi. **(A,B)** Follicles and **(C,D)** gut of females after ds*GFP* treatment. **(E,F)** Follicles and **(G,H)** gut of females after ds*NlSLC26A10* treatment. **(D’,H’)** The partial enlargement of the gut. The green signal represents the location of *Nl*SLC26A10. Scale bar: 20 μm **(A–H)**; 20 μm **(D’)**; and 10 μm **(H’)**.

To detect the expression of *Nl*SLC26A10 in *N. lugens*, immunofluorescence staining was performed. The expression of *Nl*SLC26A10 was observed in the ovary (Figures 7A,B) and gut (Figures 7C,D). *Nl*SLC26A10 was more highly expressed in the ovariole pedicel. As shown, *Nl*SLC26A10 was localized on the cytomembrane (Figures 8B,D’), consistent with the bioinformatics analysis result that *Nl*SLC26A10 contained 11 transmembrane regions. After RNAi, the follicle cells expression of *Nl*SLC26A10 was attenuated by ds*NlSLC26A10* and its morphology was shriveled (Figures 8A,B,E,F). Similarly, the expression of *Nl*SLC26A10 at the gut cell membranes was weakened after ds*NlSLC26A10* treatment (Figures 8C,D,G,H).

### Effects of RNAi on Genes Related to Ovarian Development

**Figure 9:**
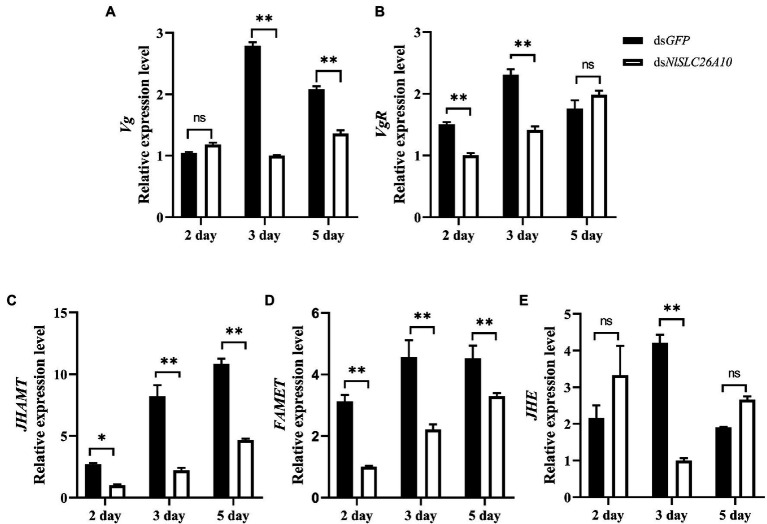
Effects of ds*NlSLC26A10* on the transcription level of genes [*Vg* **(A)**, *VgR* **(B)**, *JHAMT* **(C)**, *FAMET* **(D)**, and *JHE* **(E)**] related to ovarian development at different day post-injection of dsRNA. Data were analyzed by GraphPad Prism 2.0 and two-way ANOVA. All data are presented as the mean ± standard error. Significant difference: * means *p* < 0.05, ** means *p* < 0.01, and ns means no significance.

The changes in the transcription level of some genes are related to ovarian development after RNAi. Vitellogenin (Vg) and the vitellogenin receptor (VgR) are important in the reproductive development of insects and act key roles in ovarian development (Cong et al., 2015; Shang et al., 2018; Hu et al., 2019; Zou et al., 2020). The relative expression levels of *Vg* and *VgR* were detected after ds*NlSLC26A10* and ds*GFP* treatments. The results showed the *Vg* expression level in the ds*NlSLC26A10* treatment was markedly decreased at 3 and 5 d.p.i. (Figure 9A), and the expression level of *VgR* had a marked decrease at 2 and 3 d.p.i. (Figure 9B) compared with the ds*GFP* treatment. These indicate RNAi of *NlSLC26A10* reduces the expression of *Vg* and *VgR* in *N. lugens*.

Because the synthesis of insect Vg is generally regulated by juvenile hormones (JH; Song et al., 2014; Lyu et al., 2019; Gijbels et al., 2020), and JH plays major roles in development, ovary maturation, and reproduction in insects (Riddiford et al., 2003), genes related to JH synthesis and degradation were examined, including JHAMT (juvenile hormone acid methyltransferase), FAMET (farnesoic acid O-methyltransferase), and JHE (juvenile hormone esterase). After ds*NlSLC26A10* treatment, the expression level of *JHAMT* decreased at 2 d.p.i., and significantly decreased at 3 and 5 d.p.i., compared with the ds*GFP* treatment (Figure 9C). Similarly, the expression level of *FAMET* was significantly decreased at 2, 3, and 5 d.p.i. (Figure 9D) and *JHE* significantly decreased at 3 d.p.i. (Figure 9E). These results indicate that the RNAi *NlSLC26A10* affects the expression of Vg by regulating JH.

## Discussion

Ovarian development, spawning, and hatchability of eggs were affected by ds*NlSLC26A10*. This phenomenon may be attributed to the nutritional signals of reproduction. The normal development of oocytes is important for insects. At present, there have been a large number of studies that verified the role of Vg and VgR in the reproduction and development of insects. Vg participates in the development of the embryo, providing nutrition and promoting oocyte development (Sappington and Raikhel, 1998; Tufail and Takeda, 2008). Knockdown of *NlVg* inhibits oocyte growth in the ovarioles, leads to oocyte abnormal development, and females fail to produce offspring (Shen et al., 2019). In the bedbug *Cimex lectularius*, the downregulation of *ClVg* expression causes ovary atrophy and inhibits reproduction (Moriyama et al., 2016). Vg is usually synthesized in fat bodies, and VgR is responsible for transferring Vg into the oocyte (Schneider, 1996). In *Bactrocera dorsalis*, the lack of VgR hinders ovary development (Cong et al., 2015). Injection with ds*NlVgR* inhibits spawning and ovary development of *N. lugens* (Lu et al., 2015). In *Agasicles hygrophila*, ds*VgR* injection sharply decreases its fecundity and the ovariole is shortened (Zhang et al., 2019). In *Aphis citricidus*, silencing the *Vg-* and *VgR*-associated genes leads to delayed embryonic development (Shang et al., 2018). TOR (Target of rapamycin) is a signal molecule, which is responsible for the regulation of cell growth in eukaryotes. In the mosquito *Aedes aegypti*, *AaTOR* knockdown reduces the *Vg* gene expression level, thus inhibiting egg development (Hansen et al., 2004). In *N. lugens*, adults injected with ds*TOR* have lower fecundity as affected by Vg (Zhai et al., 2015). *NLInR1* and *NLInR2* knockdown in *N. lugens* reduce the expression of *Vg* and *VgR* and hamper ovarian development (Liu et al., 2020). Similar results were observed in our experiments. RNAi of *NlSLC26A10* resulted in a significant expression decrease for *Vg* and *VgR*. We speculated that downregulated *NlSLC26A10* expression resulted in lower expression of Vg and thus follicles cannot develop. Lower expression of VgR disrupted uptake of Vg into the developing oocyte, thereby inhibited ovarian development.

Vg expression in *N. lugens* is correlated with the regulation of JH (Lu et al., 2016; Ge et al., 2017). During the synthesis and degradation of JH in insects, JHAMT is a key rate-limiting enzyme at the final step of the JH biosynthesis pathway (Nie et al., 2014). FAMET is involved in the final rate-limiting step (Nagaraju, 2007; Zhang et al., 2021), and JHE is used to degrade JH (Zhang et al., 2017). FAMET and JHE are two key enzymes in the synthesis and metabolism of JH, respectively. RNAi-mediated silence of *N. lugens jmt*N (*JHAMT*) reduces the Vg gene expression level, suppresses the maturation of oocytes, and lowers fecundity (Lu et al., 2016). In *Tribolium castaneum*, ds*JHAMT* injection reduces the *Vg* gene expression level (Parthasarathy et al., 2010; Sheng et al., 2011). RNAi of *FAMET* reduces *Vg* expression of *riocheir sinensis* and *Macrobrachium rosenbergii* (Qian and Liu, 2019; Chen et al., 2021). In *Apis mellifera*, injection with ds*AmJHE-like* significantly reduces *Vg* transcript levels (Lourenço et al., 2019). Regulation of downstream or upstream genes of JH also affects JH level and Vg expression. Methoprene-tolerant (Met) is a universal JH receptor. Silencing *GdMet* inhibits the expression of *JHE* and *Vg*, causing reproductive diapause of *Galeruca daurica* (Ma et al., 2021). Adenylyl cyclase (AC) plays a role in cell signaling processes. Silencing *NlAC9* (the *N. lugens* AC *like-9* gene) reduces JH concentration, reduces *Vg* expression, and decreases the number of eggs laid of *N. lugens* (Ge et al., 2017). In the present study, we found *JHAMT*, *FAMET*, and *JHE* expression were significantly decreased by ds*NlSLC26A10* treatment. Combining the above results that *Vg* expression and ovarian development were affected by ds*NlSLC26A10*, this indicates RNAi of *NlSLC26A10* results in the decrease of JH levels and then leads to the decrease of *Vg* expression and hinders the ovarian development of *N. lugens*.

Even though the function of SLC26A10 has not been reported yet, considering SLC26A10 is an anion exchanger and its high expression in the ovary, the relation of *NlSLC26A10* to the phenomena found at the present study might be explained as follows. (1) RNAi of *NlSLC26A10* induces ion homeostasis, thus weaken the functions of some ion-dependent upstream regulator of JH expression. (2) RNAi of *NlSLC26A10* affects the anions secretion and absorption of oocytes in *N. lugens*, obstructing the transport of substances related to ovarian development, then hinders the maturation of the ovary. An example in the Indian white shrimp *Penaeus indicus* is as: a solute carrier, *SLC15A4*, transports amino acids to target regions to help endocrine signaling to stimulate faster ovarian maturation (Saikrithi et al., 2020). (3) After *NlSLC26A10* is silenced, its STAS domain is destroyed; then, the interaction protein–protein interaction and ion homeostasis on the oocyte surface would be disrupted, consequently damage the biological function of the oocyte, and hinder ovarian development, because the STAS domain is essential in intracellular transport and protein–protein interactions, and its mutations cause the inefficient transport of sulfate into the cells (Shibagaki and Grossman, 2006).

After ds*NlSLC26A10* treatment, the number of YLSs entering the oocytes was also lower. RNAi of *NlSLC26A10* may result in the potential change of cytomembrane, thereby reducing YLSs’ movement to the ovary. From another perspective, RNAi of *NlSLC26A10* downregulates the *Vg* expression, and then affects the entry of YLSs into the ovary, because vitellogenesis indirectly affects YLSs entering the ovary (Nan et al., 2016). Considering YLSs are primary symbionts and provide nutrients for *N. lugens* (Pang et al., 2012; Ge et al., 2016), the decrease in YLSs number in mature oocytes may cause the reduced hatchability of eggs.

## Conclusion

In this study, we cloned and characterized *NlSLC26A10*. The RNAi-mediated knockdown of *NlSLC26A10* revealed negative effects on ovarian development, spawning, and hatchability of eggs in *N. lugens*. This indicates that *NlSLC26A10* plays an important role in the reproductive development of *N. lugens*. This effect may be through regulating the synthesis of JH, thereby affecting the expression of *Vg*, or through influencing the transovarial transmission of YLSs thereby affecting the hatching of eggs.

## Data Availability Statement

The datasets presented in this study can be found in online repositories. The names of the repository/repositories and accession number(s) can be found in the article/**Supplementary Material**.

## Author Contributions

RZ and YX: conceptualization. RZ, JJ, YL, and JY: investigation. RZ: validation. YX and XY: supervision. RZ: writing–original draft. YX: writing—review and editing. All authors contributed to the article and approved the submitted version.

## Funding

This work was supported by grants from the National Natural Science Foundation of China (31871961, U21A20223, and 31501632), Zhejiang Provincial Key R&D Project (2019C02015 and 2022C02047), Natural Science Foundation of Zhejiang Province (LY22C140007), and Fundamental Research Funds for the Provincial Universities of Zhejiang (2020YW14).

## Conflict of Interest

The authors declare that the research was conducted in the absence of any commercial or financial relationships that could be construed as a potential conflict of interest.

## Publisher’s Note

All claims expressed in this article are solely those of the authors and do not necessarily represent those of their affiliated organizations, or those of the publisher, the editors and the reviewers. Any product that may be evaluated in this article, or claim that may be made by its manufacturer, is not guaranteed or endorsed by the publisher.

## Footnotes

^1^ http://web.expasy.org/compute_pi/

^2^ http://www.cbs.dtu.dk/services/SignalP/

^3^ http://www.cbs.dtu.dk/services/TMHMM

^4^ https://www.ebi.ac.uk/Tools/hmmer/

^5^ https://www.ncbi.nlm.nih.gov/
