# Supplementary material for: Molecular Characterization and RNA Interference Analysis of SLC26A10 From Nilaparvata lugens (Stål)
Source: Front Physiol. 2022 Mar 17;13:853956. doi: 10.3389/fphys.2022.853956 (PMC8969416; doi:10.3389/fphys.2022.853956)
Supplement: Supplementary file 1 [file Data_Sheet_1.docx]

Supplementary Material

## Supplementary Table

| **Primers** | **Primer Sequence (5'–3')** | **Production length** |
| --- | --- | --- |
| **For cDNA cloning** |  |  |
| *NlSLC26A10*-F | GACGAGATGGCAGATGAAAGTGAT | 2297bp |
| *NlSLC26A10*-R | CCTGTATGTTGTTGAAGTTGTGAAA |  |
| **For qPCR** |  |  |
| *Nl18S*-qF | GTAACCCGCTGAACCTCC | 170bp |
| *Nl18S*-qR | GTCCGAAGACCTCACTAAATCA |  |
| *NlSLC26A10*-qF | TTTCTGGGAACATCACGACACATA | 147bp |
| *NlSLC26A10*-qR | ACTTTGTTCGGCTGCGTATGGTAA |  |
| *NlJHAMT*-qF | GAGCACTGGTTTCAAAGTAACGGACTG | 101bp |
| *NlJHAMT*-qR | ACGGGTTCACTGCATTCAGAGACTC |  |
| *NlFAMET* -qF | GCAAAGTCAGCAATCCGCAAGAAC | 200bp |
| *NlFAMET* -qR | ACACCGTAGTGGGTGACAACGAATG |  |
| *NlJHE-*qF | AAGTAACTGGCAGATTCAACC | 200bp |
| *NlJHE-*qR | CTCGAATAGATGTGCTGCAGG |  |
| *NlVg*-qF | TTCCGTTTGCAGCCACCTATG | 154bp |
| *NlVg*-qR | CTGCTGCTGCTGCTTCTGTCA |  |
| *NlVgR*-qF | AGGCAGCCACACAGATAACCGC | 136bp |
| *NlVgR*-q | AGCCGCTCGCTCCAGAACATT |  |
| **For dsRNA synthesis** |  |  |
| T7-*GFP*-dsF | GGATCCTAATACGACTCACTATAGGGATACGTGCAGGAGAGGAC | 350 bp |
| T7-*GFP*-dsR | GGATCCTAATACGACTCACTATAGGGCAGATTGTGTGGACAGG |  |
| T7-*NlSLC26A10*-dsF | GGATCCTAATACGACTCACTATAGGACATATAGCTGTTGGACT | 591bp |
| T7-*NlSLC26A10*-dsR | GGATCCTAATACGACTCACTATAGGGAAGTCTTGCTAGTTG |  |

**Supplementary Table 1.** The primers used in this study.

## Supplementary Figures

**
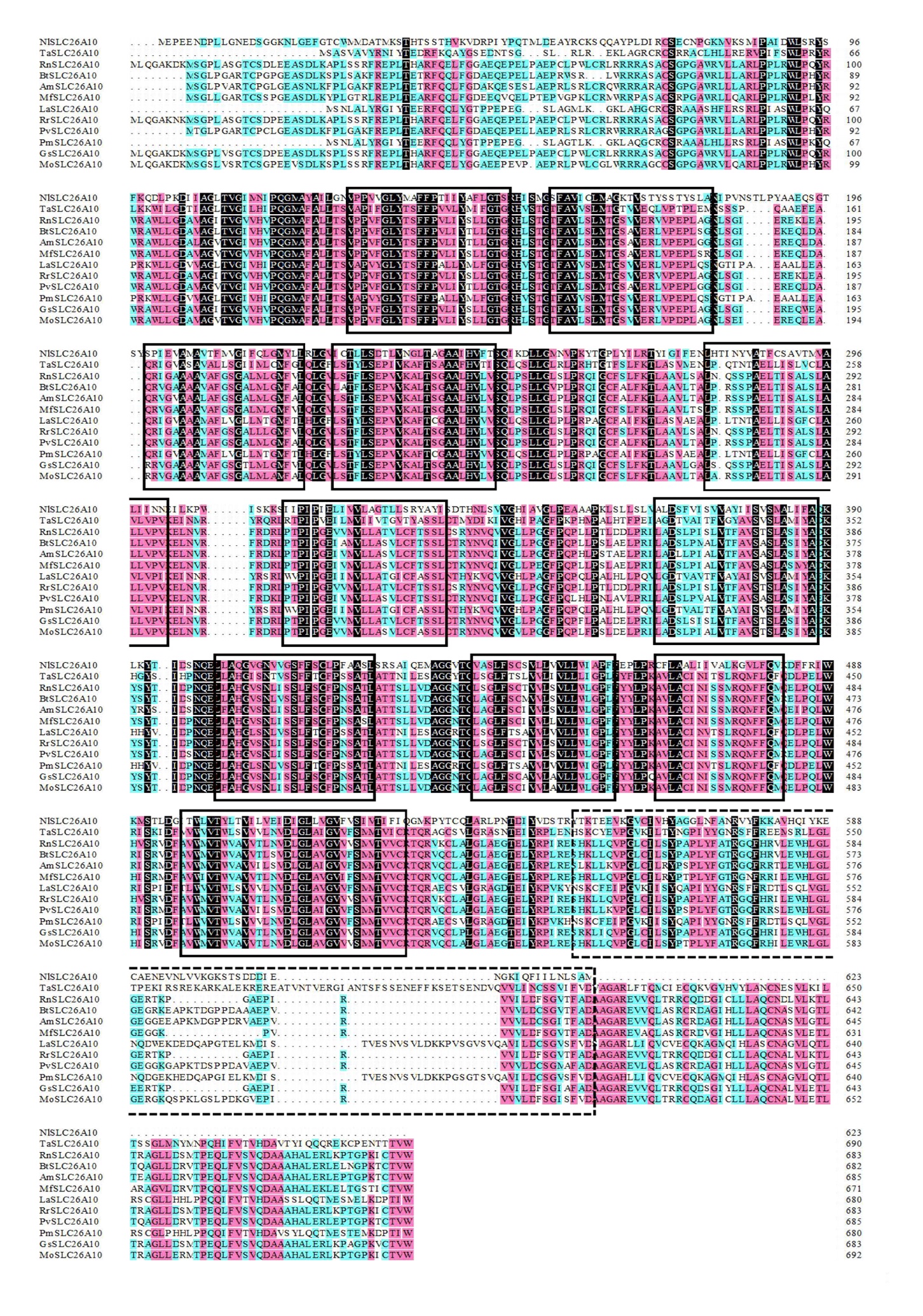
**

**Supplementary Figure S1.** Amino acid sequence comparison of SLC26A10. The domains of Sulfate_transp are shown by full-lined box and STAS with dot-lined box. *Thunnus albacares*, XP_044208539.1; *Rattus norvegicus*, NP_001128067.2; *Bos taurus*, NP_001015530.2; *Ailuropoda melanoleuca*, XP_011219081.1; *Marmota flaviventris*, XP_027781908.1; *Lacerta agilis*, XP_032993661.1; *Rattus rattus*, XP_032769493.1; *Phoca vitulina*, XP_032276801.1; *Podarcis muralis*, XP_028577161.1; *Grammomys surdaster*, XP_028607891.1; *Microtus ochrogaster*, XP_026640378.1.
